# Neuroprotective effect and possible mechanism of edaravone in rat models of spinal cord injury: a protocol for a systematic review and meta-analysis

**DOI:** 10.1186/s13643-023-02306-1

**Published:** 2023-09-26

**Authors:** Xiao-bo Wang, Long-yun Zhou, Xu-qing Chen, Ran Li, Bin-bin Yu, Meng-xiao Pan, Lu Fang, Jian Li, Xue-jun Cui, Min Yao, Xiao Lu

**Affiliations:** 1 Spine Disease Institute, Longhua Hospital, Shanghai University of Traditional Chinese Medicine, Shanghai, 200032 China; 2 Key Laboratory of Theory and Therapy of Muscles and Bones, Ministry of Education, Shanghai University of Traditional Chinese Medicine, Shanghai, 200032 China; 3 Department of Rehabilitation Medicine, The First Affiliated Hospital of Nanjing Medical University, Nanjing, 210029 Jiangsu China; 4 Department of Otolaryngology, Jiangsu Province Hospital of Chinese Medicine, Affiliated Hospital of Nanjing University of Chinese Medicine, Nanjing, 210029 Jiangsu China; 5 Traditional Chinese Medicine Hospital of LuAn, Luan, 237006 Anhui China

**Keywords:** Edaravone, Spinal cord injury, Neuroprotective effect, Rat, Protocol, Systematic review

## Abstract

**Background:**

Spinal cord injury (SCI) is one of the most disabling neurological conditions, afflicting thousands of human beings. Edaravone, a well-known reactive oxygen species scavenger, is expanding its new scope in field of SCI. The objective of this systematic review is to determine the neuroprotective effects and discuss the underlying mechanism of edaravone in management of SCI.

**Methods:**

The systematic review will include the controlled studies evaluating the neurological roles of edaravone on experiment rat models following SCI. The primary outcome will be the 21-point Basso, Beattie, and Bresnahan locomotor rating scale. The secondary outcomes will include the preservation of white matter areas and malondialdehyde levels. Two researchers will independently search PubMed, Embase, Web of Science, Scopus and Cochrane Library from their inception date. Following study selection, data extraction, and assessment of methodological quality in included studies using the SYRCLE’s RoB tool, data from eligible studies will be pooled and analyzed using random-effects models with RevMan 5.3 software. In case of sufficient data, subgroup analyses with respect to species, age, gender, injury characteristics, or administration details will be carried out to explore the factors modifying efficacy of edaravone. For exploring the appropriate dose of edaravone, a network meta-analysis approach will be conducted based on the Bayesian method. Importantly, the proposed mechanisms and changes of related molecules will be also extracted from included studies for comprehensively investigating the mechanisms underlying the neuroprotective effects of edaravone.

**Discussion:**

In this study, we aim to quantitatively analyze the role of edaravone in locomotor recovery and tissue damage in SCI rat model. The efficacy of edaravone in distinct scenarios will be investigated by subgroup analyses, and we expect to predict the candidate dose that offers a superior treatment effect using network meta-analyses. Moreover, a comprehensive framework regarding the neuroprotective mechanisms behind edaravone will be constructed via a combination of systematic and traditional review. This study will bring implications for future preclinical studies and clinical applications of SCI. Nonetheless, in light of the anticipated limitations in animal experimental design and methodological quality, the results in this review should be interpreted with caution.

**Supplementary Information:**

The online version contains supplementary material available at 10.1186/s13643-023-02306-1.

## Background

Spinal cord injury (SCI) is a devastating neurological disorder with a high rate of disability that may have lifelong effects on the patients, such as altered sensation in limbs, loss of motor function and even death [1–3]. Worldwide, approximately 20–30 million people suffer from varying degrees of SCI, with over 700,000 new events of traumatic SCI annually [4, 5]. However, there are still few definitive treatment strategies available.

For neural tissue under physiological condition, a small number of oxidants primarily derived from adenosine triphosphate synthesis in mitochondria is neutralized by endogenous superoxide dismutase to maintain homeostasis of free radicals [6, 7]. However, this balance is disrupted with irreversible consequences after SCI. The microcirculatory dysfunction and mitochondria disorder will promptly arise following the initial trauma to spinal cord, leading to the accumulation and large generation of reactive oxygen species (ROS) [8, 9]. The oxidative stress can be preserved over the long term and widely involves in the pathological events of secondary injury, causing a sequential damage cascade and disturbing the microenvironment for tissue remodeling [10]. Therefore, regulation of excessive oxidative stress represents a promising therapeutic strategy for SCI.

Edaravone, a well-known synthetic ROS scavenger, is widely used in the clinical management of cerebral hemorrhage and infarction [11]. Due to the reliable safety and efficacy, it has been approved for the treatment of amyotrophic lateral sclerosis in Japan and the United States [12]. Recently, emerging studies have evaluated the application of edaravone in extended areas, such as diabetes, tumor and cardiovascular diseases [13–16]. Given the critical role of ROS in SCI, edaravone is also finding its new scope in flied of SCI. A number of pre-clinical studies have revealed satisfactory efficacy of edaravone in treating this disease [17–25]. However, integrated pre-clinical evidences focused on the neuroprotective effect of edaravone on SCI is still scarce. Moreover, the mechanism underlying the neuroprotective effect of edaravone remains to be elucidated. Here, we propose a comprehensive systematic review of the current literature to assess the neuroprotective effects of edaravone in setting of experimental SCI and summarize the underlying molecular mechanisms.

## Methods

### Protocol and registration

This protocol was designed according to the Preferred Reporting Items for Systematic Reviews and Meta-Analyses Protocols (PRISMA-P). A summary of the protocol has been registered on PROSPERO: CRD42022374914. We aimed to answer the following questions: can edaravone promote the neurological function recovery and ameliorate tissue damage in rats following SCI? What is the mechanism underlying neuroprotective effect of edaravone in treating SCI?

### Eligibility criteria

#### Types of studies

The controlled studies evaluating the neurological roles of edaravone on experiment SCI in rat models will be included. The clinical studies, case reports, in vitro studies, reviews and comments will be excluded. No restrictions on the language, publication date, or publication status are specified [26].

#### Types of animal models

We will include laboratory rats of any age, sex, or strain that received traumatic SCI caused by compression or contusion. Any other methods for modelling SCI, such as non-traumatic ischemia, laceration, transection, traumatic root avulsion injury or genetic modifications, will be excluded because these models do not represent the typical injury mechanisms of human crush SCI [27].

#### Types of intervention

The assessed intervention will be edaravone administration to rats with SCI. The dosage, formulation and administration methods of edaravone will be unrestricted. Multiple treatment combinations (e.g., edaravone plus bone marrow mesenchymal stem cells) will be excluded.

#### Types of comparators

The edaravone-treated populations will be compared with the placebo or control group, which receives saline, saline with diluted dimethyl sulfoxide, vehicle, or no treatment. Studies will be excluded in the absence of placebo or control group.

#### Types of outcome measures

##### Primary outcome

The 21-point Basso, Beattie, and Bresnahan (BBB) locomotor rating scale was considered as primary outcomes. Briefly, the researchers independently documented the limb movements and walking characteristics of the study subjects in an open field at the same time, then assigned a BBB scale score for indicating basic locomotion function. The scales are scored from 0 (complete paralysis)-21 (normal locomotion), representing the grading of the animal's locomotor outcome after SCI [28]. Only the data at same time points will be used for analyses of BBB scores.

##### Secondary outcomes

The secondary outcomes will include spared white matter area and malondialdehyde levels. White matter damage is one of the main causes of motor loss after SCI, and the residual white matter is critical for the recovery of motor function in the hind limbs of animals [29]. Malondialdehyde is the end product of free radical-mediated lipid peroxidation reaction in tissues. It can not only respond to the rate and intensity of peroxidation in the organism, but also indirectly reflects the degree of tissue peroxidative damage [30]. To reduce the risk of data heterogeneity, only data obtained by the same detection method will be used in the analyses.

## Information sources and search strategy

### Information sources

The following electronic databases will be searched from their inception date: PubMed, Embase, Web of Science, Scopus, and Cochrane Library. We will also screen the reference lists of selected studies and reviews for covering the additional eligible studies not retrieved by the search. There will be no restrictions on language, publication date, or publication status.

### Search strategy

Search strategies will be constructed by combining the Medical Subject Headings terms and free-text terms related to the interested disease, intervention and animal. The key terms “spinal cord injuries”, “spinal cord injury”, “spinal cord diseases”, “spinal cord compression”, “spinal cord trauma”, “edaravone”, “edaravone”, “MCI 186”, “rats” and “rat” will be used. A preliminary search strategy for PubMed can be viewed in Additional file 1.

## Study selection and data extraction

### Procedure for study selection

**Fig. 1:**
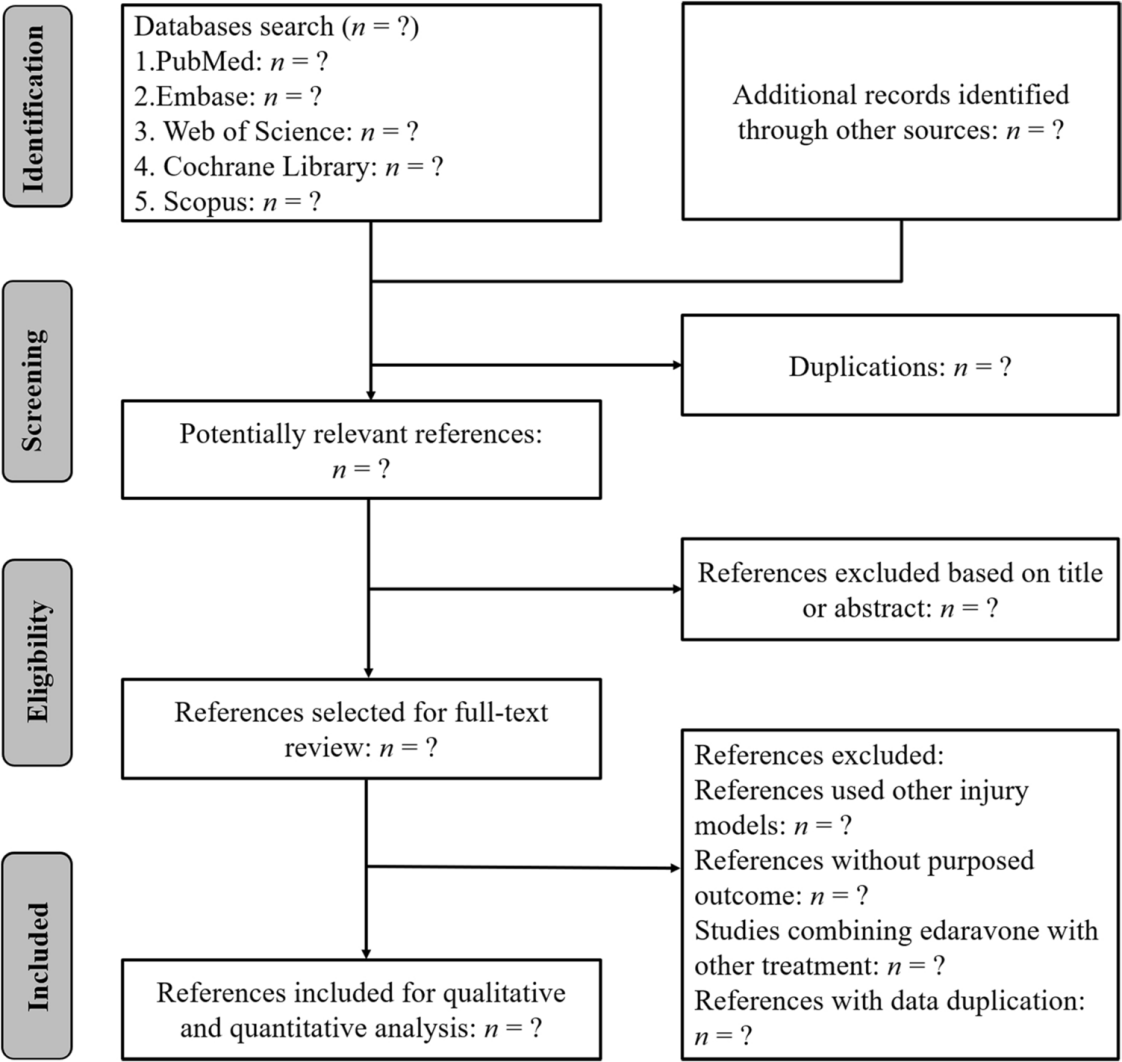
Summary of the literature identification and selection process

The results retrieved by the initial searches will be imported into NoteExpress 3.2. Following exclusion of duplicates, two reviewers will independently screen the titles and abstracts of retrieved studies to identify studies that potentially meet the inclusion criteria. Then, the full text of these potentially eligible studies will be accessed by the two reviewers independently to identify whether it could be included (Fig. 1). A senior author will involve to make the final decision when there is a disagreement between two researchers.

### Data extraction

Two reviewers will independently perform data extraction and records of study information. The following data will be extracted: name of the first author, publication year, study population (strain, number, sex, age, and weight), model characteristics (method of model, injury level), intervention details (dosage, administration route, timing, and times), and measurements. Moreover, the proposed mechanisms and changes of related molecules will be also extracted from included studies for investigating the neuroprotective mechanism behind edaravone [26, 31]. In studies involving multiple intervention groups, only data from edaravone and negative control groups will be extracted for analyses. If study data are not represented by numerical values but in graphs, we will use the GetData Graph Digitizer 2.26 (http://getdata-graph-digitizer.com) to extract the corresponding statistics from the graphs. If the needed data are missing, we will initiate contact with the authors and await a response for a two-week response period. Within waiting period, the original authors will be contacted weekly. If there is no response, the related study will be included for qualitative analyses only, but not be considered for quantitative analyses.

### Risk of bias assessment

The SYstematic Review Centre for Laboratory animal Experimentation risk of bias (SYRCLE’s RoB) tool will be used to assess the methodological quality of the included studies and to determine the intrinsic bias of the studies [32]. The checklist includes 10 items regarding selectivity bias, implementation bias, measurement bias, missed visit bias, reporting bias, and other biases. According to the SYRCLE’s risk of bias tool, two investigators independently scored those domains in each study with “yes”, “no” or “unclear”, representing a low, high, and unclear risk of bias, respectively. Any discrepancy will be resolved by consensus and the involvement of a third collaborator [33].

### Data synthesis

Summaries and analyses of data from included studies will be performed using RevMan 5.3 software (provided by the Cochrane Collaboration). When at least 3 eligible studies are identified, a meta-analysis comparing edaravone group to control group will be conducted. In accordance with the recommendations of the Cochrane Handbook for Systematic Reviews of Interventions, for studies with multiple intervention groups, combining those groups will be performed to enable a single pair-wise comparison. The mean, standard deviation, as well as the sample size of animals in each group will be used for comparisons. Summaries of intervention effects for each study will be represented as weighted mean differences or standardized mean differences with 95% confidence intervals. For the assessment of heterogeneity of included studies, we employ the *P* value in the χ2 test and Cochrane’s *I*^2^. Heterogeneity is presumed in the event that the *P* value is less than 0.10, and is considered to be high when the *I*^2^ value is more than 50%. A random effects model will be used to account for anticipated heterogeneity due to the exploratory nature of animal studies. A *P* value less than 0.05 will be considered statistically significant. Linear graphs will be constructed by GraphPad Prism software to highlight the dynamic weighted mean differences of BBB scores and dynamic BBB score improvements in both groups.

If data are available to create at least two subgroups (≥ two studies in each subgroup), subgroup analyses by species, age, sex, injury type, injury level, dose, intervention duration, or administration route will be carried out to explore the factors modifying on BBB scores. Meanwhile, sensitivity analyses on BBB scores are planned to be performed by excluding either all single studies, non-investigator-blind studies or other type of studies, which is intended to test the robustness of our findings and analyze the heterogeneous source. If we include at least 10 studies in a meta-analysis related to primary outcomes, funnel plots will be employed to test the potential risk of publication bias.

In addition, to explore the appropriate dose of edaravone, a network meta-analysis approach will be conducted according to the Bayesian method using Stata 12.0 (StataCorp LP, College Station, TX, USA). This approach intends to simultaneously compare the therapeutic effects between different doses using both direct and indirect treatment comparisons. We will calculate the probabilities of efficacy for each treatment dose, and subsequently rank the treatments based on surface area under the cumulative ranking curve.

## Discussion

In spite of enormous funding and numerous invested into treatments for SCI, an important challenge is the limited therapeutic options in this field [34]. Emerging pre-clinical studies have implied a neuroprotective role of edaravone in treating SCI. Here, we plan to initiate a systematic review in this filed, aiming to answer the question whether edaravone can facilitate the neurological function recovery in rats following SCI, and summarize the neuroprotective mechanism behind this classical agent.

To our knowledge, this prespecified review will be the first meta-analysis quantitatively analyzing the neurological roles of edaravone in SCI. The strengths and expected impact of our study are as follows. Firstly, our meta-analysis is planned to focus on the dynamic changes of BBB scores following edaravone intervention, and dealt with the tissue protective and antioxidant effects of edaravone in setting of SCI. If the outcomes reflecting the function, tissue structure and molecular changes are superior in rats treated with edaravone, it will contribute to a comprehensive understanding for the efficacy of edaravone in rats with SCI, and may reveal the clinical application prospect of this agent. Secondly, we intend to perform subgroup analyses with respect to species, age, gender, injury characteristics, or administration details. Meanwhile, network analyses based on the Bayesian method will be carried out to predict the dose with a superior neuroprotective effect. This design is beneficial to investigate the responders’ profiles and administration details of edaravone. We expect to provide optimized parameters for the implementation of future pre-clinical studies and offer a reference for the potential clinical trials. Finally, our study will propose comprehensive knowledge framework concerning the mechanisms underlying the emerging neuroprotective effects of edaravone in SCI via a combination of systematic and traditional review, and construct a schematic illustration of those mechanisms. The systematic mechanism constructed by our review will produce guidance for the basic research focusing on the in-depth molecular mechanism of edaravone in treating SCI, and may enrich evidence for evaluation of its clinical transformation. Generally, understanding the efficacy, administration details and pharmacological mechanism of certain agent is important for carrying out further pre-clinical study and assessing the prospect of its clinical application. Our study will perform a comprehensive analysis on those parameters for edaravone. Greater knowledge mapping of edaravone will facilitate basic research into SCI and may result in a new clinical therapy for this disease.

There are several predictable limitations in our study. Firstly, majority of our findings will be based on the analyses of BBB scores. This scale is a well-documented tool, while the accuracy of this outcome is limited by the assessor subjectivity [28, 35]. Given this, analyses on dynamic changes of BBB scores will be executed in our study. However, possibility of subjective bias in included studies may still result in misinterpretation on edaravone effects. Secondly, although we have developed a comprehensive search strategy, it is uncertain whether all relevant studies can be identified because we cannot access to all possible databases. Importantly, a potential risk of bias may be widely appeared in methodological quality of included studies. The bias in the methodological design may lead to the unreliable results in included studies and our meta-analyses.

To sum up, the currently proposed systematic review will evaluate the neuroprotective roles of edaravone in rats with SCI via multidimensional outcomes, analyze the application scenarios of edaravone, and discuss the underlying therapeutic mechanism. The results of this study will construct knowledge framework to understand the neuroprotective effect and possible mechanism of edaravone in management of SCI. We hope this study will help to guide future preclinical studies, and provide evidence for assessing the prospect of edaravone for future clinical trials and applications.

### Supplementary Information

**Additional file 1.** PubMed database search strategy.

## Data Availability

All data generated and/or analyzed in this study will be included in the final published article and its supplementary files.

## Abbreviations

BBB: Basso, Beattie, and Bresnahan
PRISMA-P: Preferred Reporting Items for Systematic Reviews and Meta-Analyses Protocols
ROS: Reactive oxygen species
SCI: Spinal cord injury
SYRCLE’s RoB: SYstematic Review Centre for Laboratory animal Experimentation risk of bias
