# Supplementary material for: Neuroprotective effect and possible mechanism of edaravone in rat models of spinal cord injury: a protocol for a systematic review and meta-analysis
Source: Syst Rev. 2023 Sep 26;12:177. doi: 10.1186/s13643-023-02306-1 (PMC10521558; doi:10.1186/s13643-023-02306-1)
Supplement: Supplementary file 1 — Additional file 1. PubMed database search strategy. [file 13643_2023_2306_MOESM1_ESM.docx]

**Additional file 1: PubMed database search strategy**

**Search Strategy**

(edaravone[MeSH Terms] OR edaravone[All Fields] OR norantipyrine[All Fields] OR norphenazone[All Fields] OR edarabone[All Fields] OR 1-Phenyl-3-methyl-5-pyrazolone[All Fields] OR 1 Phenyl 3 methyl 5 pyrazolone[All Fields] OR 3-Methyl-1-phenyl-2-pyrazolin-5-one[All Fields] OR 3 Methyl 1 phenyl 2 pyrazolin 5 one[All Fields] OR MCI 186[All Fields] OR MCI-186[All Fields] OR MCI186[All Fields] OR radicava[All Fields] OR phenylmethylpyrazolone[All Fields]) AND (spinal cord injuries[MeSH Terms] OR trauma, nervous system[MeSH Terms] OR spinal cord diseases[MeSH Terms] OR central cord syndrome[MeSH Terms] OR spinal cord compression[MeSH Terms] OR nervous system diseases[MeSH Terms] OR spinal cord trauma[All Fields] OR cord trauma, spinal[All Fields] OR cord traumas, spinal[All Fields] OR trauma, spinal cord[All Fields] OR traumas, spinal cord[All Fields] OR spinal cord traumas[All Fields] OR myelopathy, traumatic[All Fields] OR myelopathies, traumatic[All Fields] OR traumatic myelopathies[All Fields] OR traumatic myelopathy[All Fields] OR spinal cord injury[All Fields] OR cord injury, spinal[All Fields] OR cord injuries, spinal[All Fields] OR injury, spinal cord[All Fields] OR injuries, spinal cord[All Fields] OR spinal cord injuries[All Fields] OR spinal cord contusion[All Fields] OR cord contusion, spinal[All Fields] OR cord contusions, spinal[All Fields] OR contusion, spinal cord[All Fields] OR contusions, spinal cord[All Fields] OR spinal cord contusions[All Fields] OR spinal cord compression[All Fields] OR cord compression, spinal[All Fields] OR cord compressions, spinal[All Fields] OR compression, spinal cord[All Fields] OR compressions, spinal cord[All Fields] OR spinal cord compressions[All Fields] OR dorsal column injury[All Fields] OR corticospinal tract injury[All Fields] OR nervous system diseases[All Fields] OR nervous system disease[All Fields]) AND (rats[MeSH Terms] OR murinae[MeSH Terms] OR rats, inbred strains[MeSH Terms] OR rats[All Fields] OR rat[All Fields] OR rattus[All Fields] OR rats, laboratory[All Fields] OR rat, laboratory[All Fields] OR laboratory rats[All Fields] OR laboratory rat[All Fields])
